# mRNA COVID‐19 vaccine effectiveness against SARS‐CoV‐2 infection in a prospective community cohort, rural Wisconsin, November 2020 to December 2021

**DOI:** 10.1111/irv.12970

**Published:** 2022-02-18

**Authors:** Huong Q. McLean, David L. McClure, Jennifer P. King, Jennifer K. Meece, David Pattinson, Gabriele Neumann, Yoshihiro Kawaoka, Melissa A. Rolfes, Edward A. Belongia

**Affiliations:** ^1^ Marshfield Clinic Research Institute Marshfield Wisconsin USA; ^2^ Department of Pathobiological Sciences, School of Veterinary Medicine University of Wisconsin Madison Wisconsin USA; ^3^ Centers for Disease Control and Prevention Atlanta Georgia USA

**Keywords:** COVID‐19, SARS‐CoV‐2, vaccine effectiveness

## Abstract

Reduced COVID‐19 vaccine effectiveness (VE) has been observed with increasing predominance of SARS‐CoV‐2 Delta (B.1.617.2) variant. Two‐dose VE against laboratory‐confirmed SARS‐CoV‐2 infection (symptomatic and asymptomatic) was estimated using Cox proportional hazards models with time‐varying vaccination status in a prospective rural community cohort of 1266 participants aged ≥12 years. Between November 3, 2020 and December 7, 2021, VE was 56% for mRNA COVID‐19 vaccines overall, 65% for Moderna, and 50% for Pfizer‐BioNTech. VE when Delta predominated (June to December 2021) was 54% for mRNA COVID‐19 vaccines overall, 59% for Moderna, and 52% for Pfizer‐BioNTech.

## 1 BACKGROUND

Multiple studies have demonstrated high effectiveness of coronavirus disease (COVID‐19) vaccines in real‐world settings.^1^ However, some studies have found reduced vaccine effectiveness (VE) against severe COVID‐19 caused by acute respiratory syndrome coronavirus 2 (SARS‐CoV‐2) Delta (B.1.617.2) variant among persons who are immunocompromised, and against symptomatic infection at longer time since vaccination.^1^, ^2^, ^3^, ^4^, ^5^, ^6^ Furthermore, most evaluations of COVID‐19 VE have focused on prevention of medically attended SARS‐CoV‐2 infection or on effectiveness in high‐risk populations, such as healthcare workers. We conducted active surveillance in a well‐defined rural community cohort to estimate the effectiveness of messenger RNA (mRNA) COVID‐19 vaccines against symptomatic and asymptomatic laboratory‐confirmed SARS‐CoV‐2 infection.

## 2 METHODS

This analysis used data collected from an ongoing prospective community cohort study to assess SARS‐CoV‐2 infection in rural central Wisconsin, United States. Participants were enrolled November 2020 to March 2021, and actively monitored weekly (up to 52 weeks) to ascertain symptoms and identify new SARS‐CoV‐2 infections.

### 2.1 Study population

Participants were randomly sampled community‐dwelling individuals living in the Marshfield Epidemiologic Study Area (central region), a 14 zip code region in central Wisconsin that includes Marshfield and surrounding area. The population is ~53,000, and 89% receive most of their care from Marshfield Clinic Health System (MCHS).^7^ Further description of cohort eligibility are in the supporting information.

### 2.2 Weekly illness surveillance

Each week, all participants reported the absence or presence of specific symptoms. Anterior nasal swabs were self‐collected (or parent‐collected) for each qualifying illness episode. In addition, approximately half of the cohort was assigned to collect swabs weekly for the first 26 weeks. Further details on assignment to symptomatic versus weekly nasal swab collection are provided in the supporting information. A qualifying illness was a new illness onset in the past 7 days and at least one of the following symptoms: cough, fever or chills, sore throat, muscle or body aches, loss of smell or taste, shortness of breath, or diarrhea. Participants were instructed to also report any new respiratory symptoms by phone as soon as symptoms developed.

### 2.3 Defintions and data collection

Participants had laboratory‐confirmed SARS‐CoV‐2 infection if a specimen collected during surveillance was positive for SARS‐CoV‐2 by real‐time reverse transcription polymerase chain reaction (rRT‐PCR)^8^ or if they tested positive from a clinical PCR‐based test at MCHS. Dates and results of clinical SARS‐CoV‐2 tests and vaccinations were extracted from MCHS electronic health records and obtained from self‐report. Additional information regarding vaccinations, vaccine eligibility in Wisconsin, collection of demographic information and serum samples, and laboratory methods are described in the supporting information.

### 2.4 Analysis

Participant characteristics were compared across groups using chi‐square or Wilcoxon rank‐sum tests. VE against laboratory‐confirmed SARS‐CoV‐2 infection was estimated using Cox proportional hazards models with time‐varying vaccination status, respiratory sample collection frequency, and age. Person‐time at risk began at enrollment for persons aged ≥16 years or when participants aged 12–15 years became age‐eligible for vaccination (May 13, 2021 or 12th birthday after May 13, 2021), and ended December 7, 2021, date of positive SARS‐CoV‐2 infection, date of withdrawal from the study, or date of last weekly survey (study week 52), whichever occurred first. Unvaccinated person‐time was defined as time before receipt of the first dose. Vaccinated person‐time began ≥ 14 days after receipt of the second dose. Person‐time from receipt of the first dose through 13 days after the second dose, and after receipt of vaccine off‐label (before age‐eligible or mixed‐product series) was excluded, as were days following receipt of a third dose. In addition, person‐time was excluded after receipt of Johnson & Johnson (Janssen) vaccine due to low uptake in the population.

VE for any mRNA vaccine and for each mRNA vaccine product was calculated as (1–hazard ratio) × 100%; the hazard ratio represented the ratio of SARS‐CoV‐2 infections in two‐dose vaccinated to unvaccinated person‐time. VE against symptomatic SARS‐CoV‐2 infection was estimated by excluding participants with infection with no reported symptoms during the 2 weeks before and after the positive test result (asymptomatic). VE against the Delta variant was estimated by restricting person‐time at risk to the period after June 21, 2021, when > 50% of sequenced viruses in Wisconsin were Delta.^9^ Sensitivity analyses excluded persons who self‐reported or had serologic evidence of prior SARS‐CoV‐2 infection. Analyses were conducted using SAS (Version 9.4; SAS institute).

Marshfield Clinic Research Institute (MCRI)'s Institutional Review Board reviewed and approved the study. The Centers for Disease Control and Prevention (CDC) ceded research oversight to MCRI (45 C.F.R. part 46; 21 C.F.R. part 56).

## 3 RESULTS

Of 1518 cohort participants, 1266 (83%) were aged ≥ 12 years and included in this VE analysis. By the end of follow‐up, almost half (48%) received two doses of Pfizer‐BioNTech vaccine, 26% received two doses of Moderna vaccine, and 26% were unvaccinated. Older adults, females, Non‐Hispanic White participants, those who received the 2020–2021 influenza vaccine, and those who work in healthcare were more likely to be vaccinated (Table 1). Most (76%) vaccinated participants received a second dose in January to April 2021 (Figure S1). Moderna recipients tended to be older, have a preexisting medical condition, have public insurance, and longer median time since receipt of the second dose (238 [interquartile range (IQR) 223–256] days vs. 217 [IQR 182–247] days, Wilcoxon *P* < 0.001) compared with Pfizer‐BioNTech recipients.

**TABLE 1:** Characteristics of the rural central Wisconsin community cohort, aged ≥ 12 years

		Vaccination status^a^			
	All (n = 1266)	Received Moderna COVID‐19 vaccine (n = 329)	Received Pfizer‐BioNTech COVID‐19 vaccine (n = 608)	Not vaccinated (n = 329)	SARS‐CoV‐2 infection^b^ (n = 118)
Age group^c^					
12–17 years	129 (10)	0	84 (14)	45 (14)	11 (9)
18–49 years	470 (37)	91 (28)	219 (36)	160 (49)	58 (49)
50–64 years	272 (21)	76 (23)	133 (22)	63 (19)	23 (19)
≥ 65 years	395 (31)	162 (49)	172 (28)	61 (19)	26 (22)
Sex					
Female	747 (59)	188 (57)	383 (63)	176 (54)	70 (59)
Male	519 (41)	141 (43)	225 (37)	153 (46)	48 (41)
Race/ethnicity^d^					
Non‐Hispanic White	1203 (95)	321 (98)	576 (95)	306 (93)	111 (94)
Hispanic	39 (3)	4 (1)	18 (3)	17 (5)	5 (4)
Non‐Hispanic non‐White	20 (2)	3 (1)	12 (2)	5 (2)	2 (2)
Preexisting medical condition^e^					
No	547 (43)	110 (33)	281 (46)	156 (47)	55 (47)
Yes	719 (57)	219 (67)	327 (54)	173 (53)	63 (53)
Asthma	109 (9)	27 (8)	46 (8)	36 (11)	6 (5)
Cancer	31 (2)	15 (5)	10 (2)	6 (2)	3 (3)
Chronic kidney disease	26 (2)	7 (2)	13 (2)	6 (2)	1 (1)
COPD	31 (2)	11 (3)	13 (2)	7 (2)	3 (3)
Hypertension	307 (24)	115 (35)	142 (23)	50 (15)	17 (14)
Immunocompromised	47 (4)	15 (5)	20 (3)	12 (4)	5 (4)
Serious heart condition	72 (6)	34 (10)	24 (4)	14 (4)	5 (4)
Type 2 diabetes	108 (9)	41 (12)	48 (8)	19 (6)	9 (8)
Obese	471 (37)	136 (41)	208 (34)	127 (39)	51 (43)
Receipt of 2020–2021 influenza vaccine					
No	382 (30)	63 (19)	125 (21)	194 (59)	46 (39)
Yes	884 (70)	266 (81)	483 (79)	135 (41)	72 (61)
Health insurance type^d^					
Private	804 (64)	169 (51)	425 (70)	210 (64)	83 (71)
Public	435 (34)	156 (48)	173 (29)	106 (33)	31 (27)
None	21 (2)	4 (1)	7 (1)	10 (3)	3 (3)
Occupation/Industry^f^					
Healthcare/Social services	241 (36)	45 (30)	166 (51)	30 (16)	20 (25)
Manufacturing	78 (12)	15 (10)	25 (8)	38 (20)	7 (9)
Education	75 (11)	33 (22)	23 (7)	19 (10)	13 (16)
Retail	49 (7)	5 (3)	22 (7)	22 (12)	6 (8)
Other service	38 (6)	8 (5)	21 (6)	9 (5)	3 (4)
Other industry	186 (28)	46 (30)	68 (21)	72 (38)	30 (38)
Frequency of respiratory sample collection^g^					
Weekly swabbing	635 (50)	174 (53)	295 (49)	166 (50)	67 (57)
Swabbing with qualifying illness	631 (50)	155 (47)	313 (51)	163 (50)	51 (43)
Prior SARS‐CoV‐2 infection^h^					
No	1088 (86)	292 (89)	523 (86)	273 (83)	114 (97)
Yes	178 (14)	37 (11)	85 (14)	56 (17)	4 (3)

*Note*: Data are no. (%) of participants.

Abbreviations: COPD, chronic obstructive pulmonary disease; COVID‐19, coronavirus disease 2019; SARS‐CoV‐2, severe acute respiratory syndrome coronavirus 2.

^a^ Documented receipt of two doses before the time of censoring.

^b^ New laboratory‐confirmed SARS‐CoV‐2 infections occurring during the follow‐up period (November 3, 2020 to December 7, 2021) among two‐dose vaccinated or unvaccinated participants.

^c^ Age as of end of follow‐up (or censoring).

^d^ Data missing or unknown; % based on those with data.

^e^ Preexisting medical condition includes self‐report of asthma, cancer, chronic kidney disease, COPD, hypertension, immunocompromised state, serious heart condition, and type 2 diabetes, and obesity defined as BMI ≥ 30 calculated from self‐reported height and weight.

^f^ Percent among employed participants; based on the North American Industry Classification System (NAICS) grouping of 20 large industry sectors (https://www.census.gov/naics/).

^g^ Participants assigned to swab weekly for the first 26 weeks regardless of symptoms and with a qualifying illness after study week 27 or to swab with a qualifying illness for the duration of follow‐up. A qualifying illness was an illness with new onset in the past 7 days and at least one of the following symptoms: cough, fever or chills, sore throat, muscle or body aches, loss of smell or taste, shortness of breath, or diarrhea.

^h^ Evidence from enzyme‐linked immunosorbent assays (ELISA) targeting SARS‐CoV‐2 receptor‐binding domain and spike protein conducted at the Influenza Research Institute at University of Wisconsin‐Madison on serum samples collected at enrollment, molecular SARS‐CoV‐2 test results from study or clinic testing, and self‐report at study enrollment.

Between November 3, 2020 and December 7, 2021, 118 (9%) SARS‐CoV‐2 confirmed infections were documented during follow‐up; 6 were asymptomatic (2 received Pfizer‐BioNTech), 4 were previously infected (all unvaccinated) and 51 (43%) were vaccinated (29% received Pfizer‐BioNTech, 14% received Moderna). Mean age of those infected was 47.7 years, and 59% were female (Table 1). Among unvaccinated participants with infection, 11 (16%) sought care, and 4 (6%) were hospitalized. Among vaccinated participants with infection, 4 (8%) sought care, and 1 (2%) was hospitalized. Median time from receipt of the second dose to infection was 215 (IQR 163–241) days.

**FIGURE 1:**
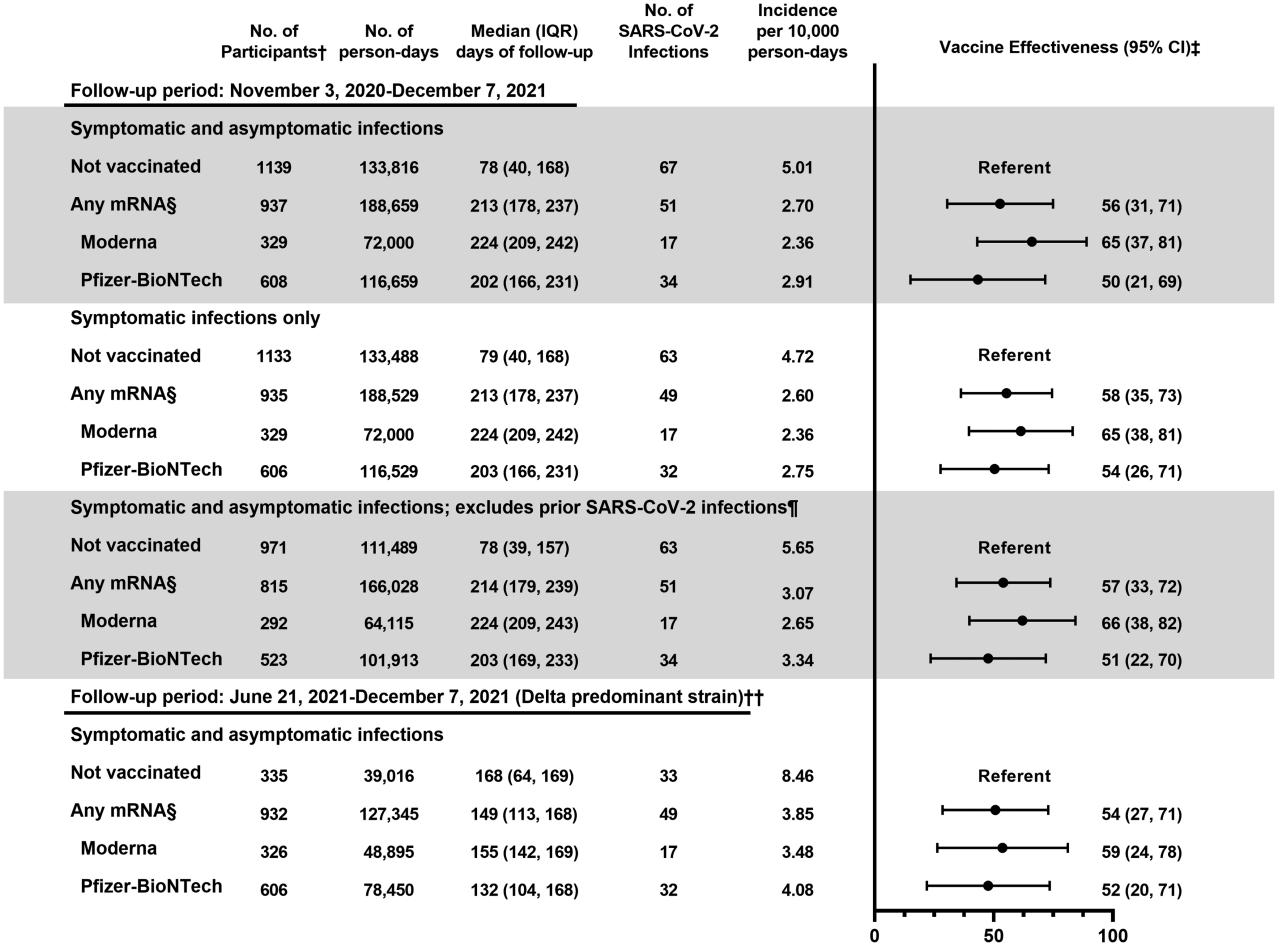
Effectiveness of COVID‐19 vaccines against SARS‐CoV‐2 infection in a rural community, Wisconsin, November 3, 2020 to December 7, 2021. †Participants can contribute both unvaccinated and vaccinated time during the follow‐up period. ‡Estimated from Cox proportional hazards model with time‐varying vaccination status, respiratory sample collection frequency, and age (modeled as natural cubic spline with five knots based on percentiles). §Vaccinated defined as ≥ 14 days after receipt of the second dose of any mRNA vaccine. ¶Defined based on evidence from enzyme‐linked immunosorbent assays (ELISA) targeting SARS‐CoV‐2 receptor‐binding domain and spike protein conducted at the Influenza Research Institute at University of Wisconsin‐Madison on serum samples collected before enrollment, molecular SARS‐CoV‐2 test results from clinic testing before enrollment, and self‐report at study enrollment. ††Delta variant identified in > 50% of samples sequenced in Wisconsin after June 21, 2021; 97% of 29 viruses sequenced from samples collected from study participants between June 21, 2021 and October 7, 2021 were Delta variant. Participants with SARS‐CoV‐2 infection between November 3, 2020 and June 20, 2021 were excluded

VE of mRNA vaccines against laboratory‐confirmed infection (symptomatic and asymptomatic) was 56% (95% confidence interval [CI] 31–71), 65% (95% CI 37–81) for Moderna, and 50% (95% CI 21–69) for Pfizer‐BioNTech (Figure 1). VE estimates were similar against symptomatic infections, when prior infections were excluded, and when restricted to the period when Delta predominated (Figure 1).

## 4 DISCUSSION

In this prospective rural community cohort with active illness surveillance, mRNA vaccines were 56% effective against symptomatic and asymptomatic SARS‐CoV‐2 infection. VE estimates were lower than reported estimates from clinical trials and observational studies based on clinical testing conducted soon after vaccines became available.^6^, ^10^, ^11^

Our findings of lower VE against SARS‐CoV‐2 infection during a period with increased circulation of Delta is consistent with previous studies among healthcare workers, nursing home residents, and in retrospective, population‐based cohort studies.^2^, ^3^, ^4^, ^5^, ^12^ Waning of vaccine protection is also possible, as lower VE was observed with longer time since vaccination in several studies.^2^, ^3^, ^13^ However, assessment of the contribution of waning immunity and the Delta variant to the observed reduced vaccine protection is complicated by the local increase in Delta circulation coinciding with the period when most cohort members were > 5 months from receipt of their second dose. Further studies with additional follow‐up time after vaccination for all ages are needed to better understand the impact of waning protection and the importance of booster doses.

This study had several limitations. Relatively few cases occurred during the follow‐up period with most vaccinated cases occurring when Delta predominated. The small sample size led to wide CIs and limited our ability to control for potential confounding factors in VE estimates such as preexisting conditions, occupation, and behaviors, which may be associated with vaccination status, vaccine product received, and infection risk. Finally, the study population is largely non‐Hispanic White and from a single rural community in central Wisconsin so findings may not be generalizable to other rural communities or other racial and ethnic groups.

Strengths of this study include active follow‐up of participants for new illness that included weekly respiratory samples collection for SARS‐CoV‐2 testing for half of the participants during most of the follow‐up period. Weekly surveillance combined with clinical SARS‐CoV‐2 test results available from linked health records allowed comprehensive capture of SARS‐CoV‐2 infections. Second, MCHS's data exchange with the Wisconsin Immunization Registry allowed more accurate classification of vaccination status over time and product received. Third, our analysis included adolescents and rural community members, who have been underrepresented to date. Finally, prior SARS‐CoV‐2 infections were captured by self‐report and serologic testing, reducing the potential for biased VE estimates.

This study demonstrates that two doses of mRNA vaccine reduce the risk of SARS‐CoV‐2 infection. However, vaccinated persons continue to be at risk for infection in the community, serving as a reminder of the importance of layered prevention measures to break chains of transmission. A booster dose may help increase protection among vaccinated persons, but efforts to increase uptake are essential. Increasing uptake of the primary vaccine series in rural areas, where there is greater hesitancy to receive COVID‐19 vaccine and the burden of COVID‐19 and associated mortality has been higher than in urban areas, should be a priority.^14^, ^15^ As SARS‐CoV‐2 continues to circulate and evolve and the COVID‐19 vaccination program matures, continued monitoring of COVID‐19 VE in the general population is needed.

## AUTHOR CONTRIBUTIONS

**David L McClure:** Conceptualization; formal analysis; methodology. **Jennifer P King:** Conceptualization; methodology; project administration. **Jennifer K Meece:** Conceptualization; methodology; project administration; supervision. **David Pattinson:** Conceptualization; data curation; methodology. **Gabriele Neumann:** Conceptualization; methodology; project administration; supervision. **Yoshihiro Kawaoka:** Conceptualization; funding acquisition; methodology; supervision. **Melissa A Rolfes:** Conceptualization; investigation; methodology; project administration; supervision. **Edward A Belongia:** Conceptualization; methodology; supervision.

## CONFLICT OF INTEREST

All authors report no potential conflicts.

## DISCLAIMER

The findings and conclusions in this report are those of the authors and do not necessarily represent the official position of the US Centers for Disease Control and Prevention.

### PEER REVIEW

The peer review history for this article is available at https://publons.com/publon/10.1111/irv.12970.

## Supporting information


**Data S1.** Supporting Information

## Data Availability

The data that support the findings of this study are available upon reasonable request from the corresponding author. The data are not publicly available due to privacy or ethical restrictions.
