# Supplementary material for: mRNA COVID‐19 vaccine effectiveness against SARS‐CoV‐2 infection in a prospective community cohort, rural Wisconsin, November 2020 to December 2021
Source: Influenza Other Respir Viruses. 2022 Feb 18;16(4):607–12. doi: 10.1111/irv.12970 (PMC9111813; doi:10.1111/irv.12970)

**mRNA COVID-19 vaccine effectiveness against SARS-CoV-2 infection in a prospective community cohort, rural Wisconsin, November 2020 to December 2021**

Huong Q McLean^1^, David L McClure^1^, Jennifer P King^1^, Jennifer K Meece^1^, David Pattinson^2^, Gabriele Neumann^2^, Yoshihiro Kawaoka^2^, Melissa A Rolfes^3^, Edward A Belongia^1^

^1^Marshfield Clinic Research Institute, Marshfield, Wisconsin, United States

^2^Department of Pathobiological Sciences, School of Veterinary Medicine, University of Wisconsin-Madison, Wisconsin, United States

^3^Centers for Disease Control and Prevention, Atlanta, Georgia, United States

**Supporting Information**

**Supplemental Methods**

***Marshfield Epidemiologic Study Area (MESA).*** The Central region of MESA is a geographically-defined population cohort of approximately 53,000 residents residing in 14 zip codes that includes Marshfield, Wisconsin and surrounding area.^1^ In this area, 89% of residents receive most of their care from Marshfield Clinic Health System (MCHS), and the population size is similar to US census estimates for the MESA zip codes.^1^ The MESA population is predominantly non-Hispanic White, and the population is older and more rural compared to the entire state.

***Cohort eligibility criteria.*** For this prospective study, we sampled and recruited MESA residents in strata defined by age group. Young children (age < 10 years) and older adults (age ≥ 70 years) were oversampled. Since enrollment was lower than expected, we opened enrollment to eligible individuals who were not sampled beginning January 2021. We excluded residents of institutional or group settings, individuals who planned to move or reside outside of the study area for ≥ 21 continuous days during the next 12 months, and participants in COVID-19 clinical trials. No exclusions were made for individuals who had had SARS-CoV-2 infection prior to enrollment, nor for individuals who had received COVID-19 vaccine doses prior to enrollment.

***Surveillance group assignment*.** Frequency of respiratory sample collection, weekly or with qualifying illness, was initially randomly assigned in 1:1 ratio from initiation of enrollment through December 28, 2020. However, because of slower than expected enrollment and concurrent increased SARS-CoV-2 circulation locally, enrollments from December 29, 2020 through January 25, 2021 were assigned to weekly respiratory sample collection. Enrollments after January 25, 2021 were assigned to respiratory sample collection with qualifying illness except children aged < 10 and Spanish-speaking participants who were assigned to weekly respiratory sample collection until enrollment targets were reached, because of low initial participation in these groups.

***Data and serum sample collection****.* At enrollment, consenting participants completed a survey on demographics, current health status, medical history, prior SARS-CoV-2 infection and exposure history, and relevant environmental and behavioral risk factors. Serum samples were collected at enrollment and approximately 12 and 24 weeks later. Additional information regarding demographics, SARS-CoV-2 clinical test dates and results, COVID-19 and influenza vaccinations, preexisting conditions, and healthcare visits were extracted from MCHS’s electronic health records. MCHS exchanges vaccination data with the Wisconsin Immunization Registry weekly. If self-report of vaccination was not documented in the electronic health records, vaccination dates, vaccine product, and location of vaccination were obtained from the participant’s COVID-19 vaccination card.

***COVID-19 vaccine roll-out in Wisconsin***. In Wisconsin, the initial phases of COVID-19 vaccine roll-out began December 16, 2020 and prioritized vaccination of frontline healthcare workers, first responders, residents of long-term care facilities, and persons aged ≥ 65 years. Vaccine became available on March 1, 2021 to those enrolled in Medicaid long-term care programs, those who work or live in congregate living facilities, public-facing essential workers, and non-frontline essential healthcare personnel. Individuals aged ≥ 16 years with certain medical conditions that have greater risk of severe infection and all individuals aged ≥ 16 years were eligible March 22 and April 5, 2021, respectively. Adolescents aged 12-15 years were vaccine eligible on May 13, 2021.

***Laboratory methods.*** Respiratory samples collected during cohort surveillance were tested by real-time reverse transcription polymerase chain reaction (rRT-PCR) for SARS-CoV-2 using the ThermoFisher Combo Kit platform at Marshfield Clinic Research Institute’s Integrated Research and Development Laboratory.^2^ A subset of positive samples (e.g., samples with cycle threshold values < 30) collected between April 12, 2021 and October 7, 2021 were sequenced using a modified ARTIC sequencing methodology (<https://artic.network/ncov-2019>). A laboratory-confirmed SARS-CoV-2 infection was defined in a participant who had a specimen collected during surveillance that was positive by rRT-PCR or a specimen collected for clinical purposes that was tested by a PCR-based assay conducted at MCHS.

Serum samples collected at the time of enrollment were tested for SARS-CoV-2 antibodies using an enzyme-linked immunosorbent assay (ELISA) that targeted the SARS-CoV-2 receptor-binding domain, the full-length spike (S1S2) protein, and nucleocapsid protein following standard procedures at the Influenza Research Institute at University of Wisconsin-Madison.^3^ Specimens collected from participants who were unvaccinated at the time of enrollment were classified as seropositive based on a Bayesian hierarchical model considering reactivity against S1S2 and the receptor-binding domain targets.

**References**

1. Kieke AL, Kieke BA, Jr., Kopitzke SL, et al. Validation of Health Event Capture in the Marshfield Epidemiologic Study Area*.* Clin Med Res 2015; 13(3-4):103-11.DOI: 10.3121/cmr.2014.1246.
2. ThermoFisher Scientific TaqPath™ COVID-19 combo kit and TaqPath™ COVID‑19 combo kit advanced* instructions for use. 2020.
3. Pattinson D, Jester P, Guan L, et al. A method to reduce ELISA serial dilution assay workload applied to SARS-CoV-2 and seasonal HCoVs. medRxiv 2021.

**Figure S1**. Number of new SARS-CoV-2 infections during the follow-up period (November 3, 2020 through December 7, 2021), and cumulative number of two-dose vaccinated participants by week.


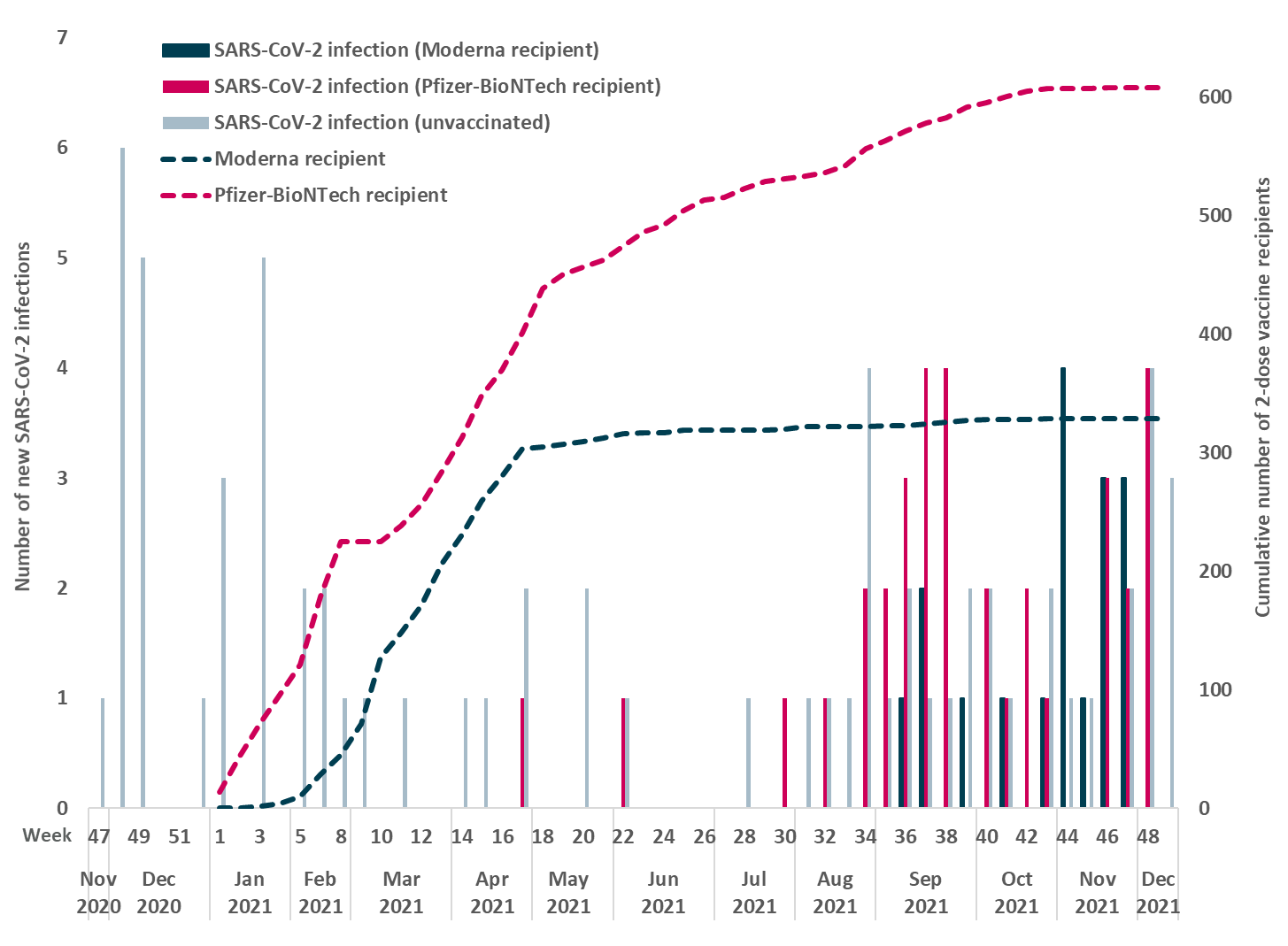

Supplement: Supplementary file 1 — Data S1. Supporting Information [file IRV-16-607-s001.docx]
